# A sustainable protocol for selective alcohols oxidation using a novel iron-based metal organic framework (MOF-BASU1)†

**DOI:** 10.1039/d3ra03058j

**Published:** 2023-08-17

**Authors:** Mahtab Yaghubzadeh, Sedigheh Alavinia, Ramin Ghorbani-Vaghei

**Affiliations:** a Department of Organic Chemistry, Faculty of Chemistry, Bu-Ali Sina University Hamedan 6517838683 Iran rgvaghei@yahoo.com ghorbani@basu.ac.ir +98-8138380647

## Abstract

The selective oxidation of active and inactive alcohol substrates is a highly versatile conversion that poses a challenge in controlling the functionality and adjustments on MOFs. On the other hand, it offers an attractive opportunity to expand their applications in designing the next generation of catalysts with improved performance. Herein, a novel iron-based MOF containing sulfonamide (MOF-BASU1) has been fabricated by the reaction of 1,3-benzene disulfonylchloride linker and FeCl_3_·6H_2_O. Based on the results, the active surface area of the synthesized MOF is large, which highlights its unique catalytic activity. Optimum conditions were reached after 0.5–2 h, with 15 mg loading of the synthesized MOF under optimal conditions. Furthermore, the turnover frequency was 18–77.6 h^−1^, which is comparable to values previously reported for this process. Overall, the high catalytic activity observed for MOF-BASU1 might be because of the obtained high surface area and the Lewis acidic Fe nodes. Furthermore, the MOF-BASU1 revealed a remarkable chemoselectivity for aldehydes in the presence of aliphatic alcohols. Overall, the high product yields, facile recovery of nanocatalysts, short reaction times, and broad substrate range make this process environmentally friendly, practical, and economically justified.

## Abstract 2

The selective oxidation of active and inactive alcohol substrates is a highly versatile conversion that poses a challenge in controlling the functionality and adjustments on MOFs.

## Graphical abstract

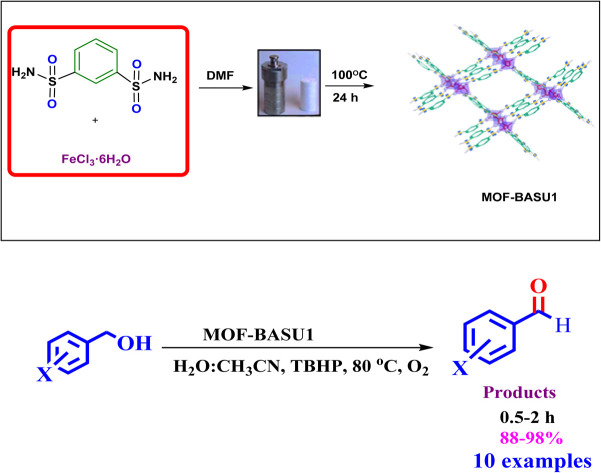

## 1. Introduction

Alcohol oxidation reactions play a vital role in industrial applications, the synthesis of synthetic intermediates, and natural products. In the pharmaceutical industry, the oxidation reactions of alcohols are used extensively.^1^ Therefore, the development of alcohol oxidations with an environmentally friendly and cost-effective strategy is one of the important and necessary concepts for the pharmaceutical and chemical industry. Alcohol oxidation is widely carried out traditionally with small organic-based reagents, such as Dess–Martin periodinane and Swern oxidation or metal-based systems, such as pyridinium chlorochromate, pyridinium dichromate, and ruthenium tetroxide.^2–5^ However, most of these have various limitations including moisture-sensitive, expensive, and not reusable. Therefore, new oxidation approaches are expected to be carried out selectively with green, cheap, and non-toxic oxidants, such as dioxygen or air in the presence of reusable catalysts.^6^

Recently, various homogeneous catalysts have played an important role owing to their efficiency and all-purpose impact on the many catalytic reactions.^7–9^ However, despite the high efficiency of homogeneous catalysts, all these reactions suffer from poor catalyst recovery. The benefits of catalyst recovery include the economic and environmentally friendly concept of special transition metals; however, the key challenges are yet to be resolved.^10–12^ One of the solutions to overcome this limitation in oxidation reactions is the immobilization of homogeneous catalysts on supports that are not chemically active. In this regard, one of the most popular materials for a wide range of applications in oxidation reactions are metal–organic frameworks (MOFs) with tunable chemical and physical properties.^13–19^

Metal organic frameworks (MOFs) are a new class of organic–inorganic hybrids that, owing to their nature, have many applications in fields, such as gas storage,^20^ catalytic processes,^21,22^ encapsulate material, super capacitors, and the absorption of heavy metals.^23^ MOFs not only have a higher level of activation and stability than other classes of porous materials, but can also change the morphology and size of cavities uncomplicatedly, and this has become an advantage in terms of separation and greater selectivity in their applications.^24–27^

Designing and synthesizing new molecular scaffolds with unique structural and biological properties that increase their capability and selectivity is an interesting challenge. Nowadays, sulfonamides, with unique features, such as strong chemical/thermal stability, are considered as new ligands by the chemical community.^28–30^ The use of sulfonamide ligands in the synthesis of metal organic frameworks can create a revolution in the MOFs field and catalysis.

**Scheme 1:**
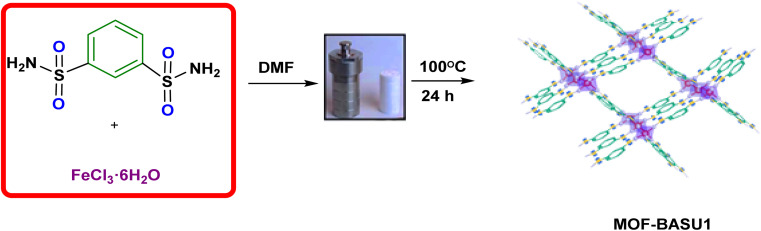
Sequential synthesis of MOF-BASU1 catalyst.

Regarding the points mentioned, this study presents the development and characterization of a new Fe-based MOF (MOF-BASU1) from the reaction of 1,3-benzenedisulfonamide (BDS) and FeCl_3_·6H_2_O (Scheme 1). The catalytic activity of the MOF-BASU1 was tested in aerobic alcohol oxidation without over oxidation. In the light of the results obtained, the high efficiency, high surface area, and easy recovery of the catalyst are strong points to justify its use. To the best of our knowledge, there are no reports on the use of a MOF-BASU1 catalyst in the synthesis of aldehyde/ketone compounds. The catalyst can usefully act as both acid and redox active sites platform. This study consistently has advantages, such as the availability of MOF, inexpensive catalyst, mild reaction conditions, reasonable yields, and simple experimental procedures. Such a potential catalytic utility of MOF-BASU1 make it quite attractive for sustainable industrial chemistry.

## 2. Experimental section

### 2.1. Instrumentation

All the chemicals, reagents, and equipment used in this study are listed in Table 1.

**Table 1:** Chemicals and equipment used in this study

Materials and equipment	Purity and brand
Iron(iii) chloride hexahydrate (FeCl_3_·6H_2_O)	Sigma–Aldrich (≥98%)
Potassium carbonate (K_2_CO_3_)	Merck (98%)
*N*,*N*-Dimethylformamide (DMF)	Merck (99.8%)
*n*-Hexane	Sigma–Aldrich (95%)
Ethanol	Sigma–Aldrich (97%)
Acetonitrile	Merck (98%)
FT-IR analysis	Shimadzu IR-470 spectrometer
EDX analysis	Numerix DXP-X10P
SEM analysis	Sigma-Zeiss microscope
XRD analysis	JEOL JDX-8030 (30 kV, 20 mA)
NMR analysis	Varian Unity Inova 500 MHz
Ultrasound cleaning bath	KQ-250 DE (40 kHz, 250 W)
Melting point measurement	Electrothermal 9100, made in UK

### 2.2. Synthesis of the MOF-BASU1 catalyst

**Scheme 2:**
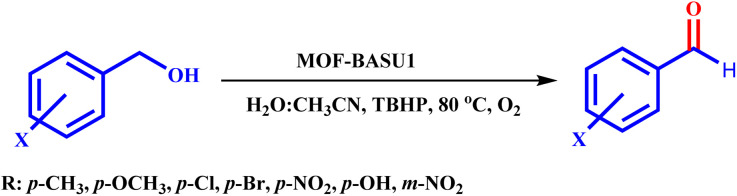
Synthesis of aldehyde derivatives in the presence of MOF-BASU1 catalyst.

MOF-BASU1 was produced by the solvothermal method. Briefly, 1 mmol of FeCl_3_·6H_2_O, 2 mmol of benzene disulfonamide (BDS), and 50 mL of DMF were poured into a beaker and dispersed in an ultrasonic bath for 10 min. Then, the mixture was stirred for 10 min at 100 °C. The resulting mixture was transferred to an autoclave and kept in an oven at 100 °C for 24 hours. Scheme 2 The obtained solution was centrifuged and the solid residues were washed several times with DMF and dried in an oven at 60 °C for 12 hours (Scheme 1).

### 2.3. General method for the synthesis of benzaldehyde derivatives

Primary benzyl alcohol analogs (1 mmol), tertiary butylhydrogen peroxide (3 mmol), and MOF-BASU1 (15 mg) were stirred at 80 °C in H_2_O : CH_3_CN (2 mL, 1 : 1, volume) for the suitable time, as illustrated in Table 3. After the reaction, the catalyst was filtered off. The carbonyl products and unreacted reactants in the reaction mixture were isolated using a facile extraction with water/ethyl acetate. The organic phase was then concentrated by full evaporation of the solvent. The residue was purified using column chromatography (*n*-hexane/EtOAc, 4 : 1, volume) yielded the desired products.

## 3. Results and discussion

### 3.1. Catalyst characterization data analysis

**Fig. 1:**
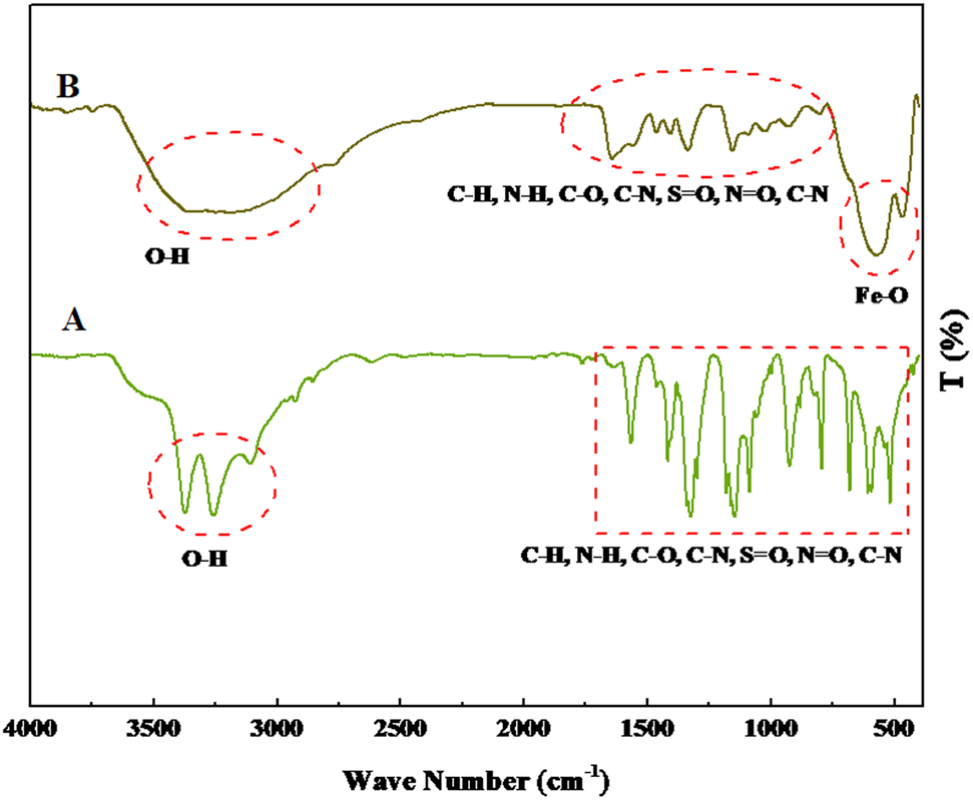
FT-IR spectra of 1,3-benzenedisulfonamide (A) and MOF-BASU1 samples (B).

The FT-IR spectra of 1,3-benzenedisulfonamide and MOF-BASU1 catalyst are shown in Fig. 1. In 1,3-benzenedisulfonamide, the absorption bands at 1145 and 1332 cm^−1^ are attributed to S=O stretching. The bands at 3344 cm^−1^ and 3248 cm^−1^ correspond to the NH_2_ stretching vibrations. After the synthesis of MOF-BASU1, a broad absorption band was observed at 3000–3500 cm^−1^ related to the interaction of amine groups and iron in the studied structure. Moreover, the absorption band at 526 cm^−1^ is associated with the Fe–O stretching in MOF-BASU1 (Fig. 1B).

**Fig. 2:**
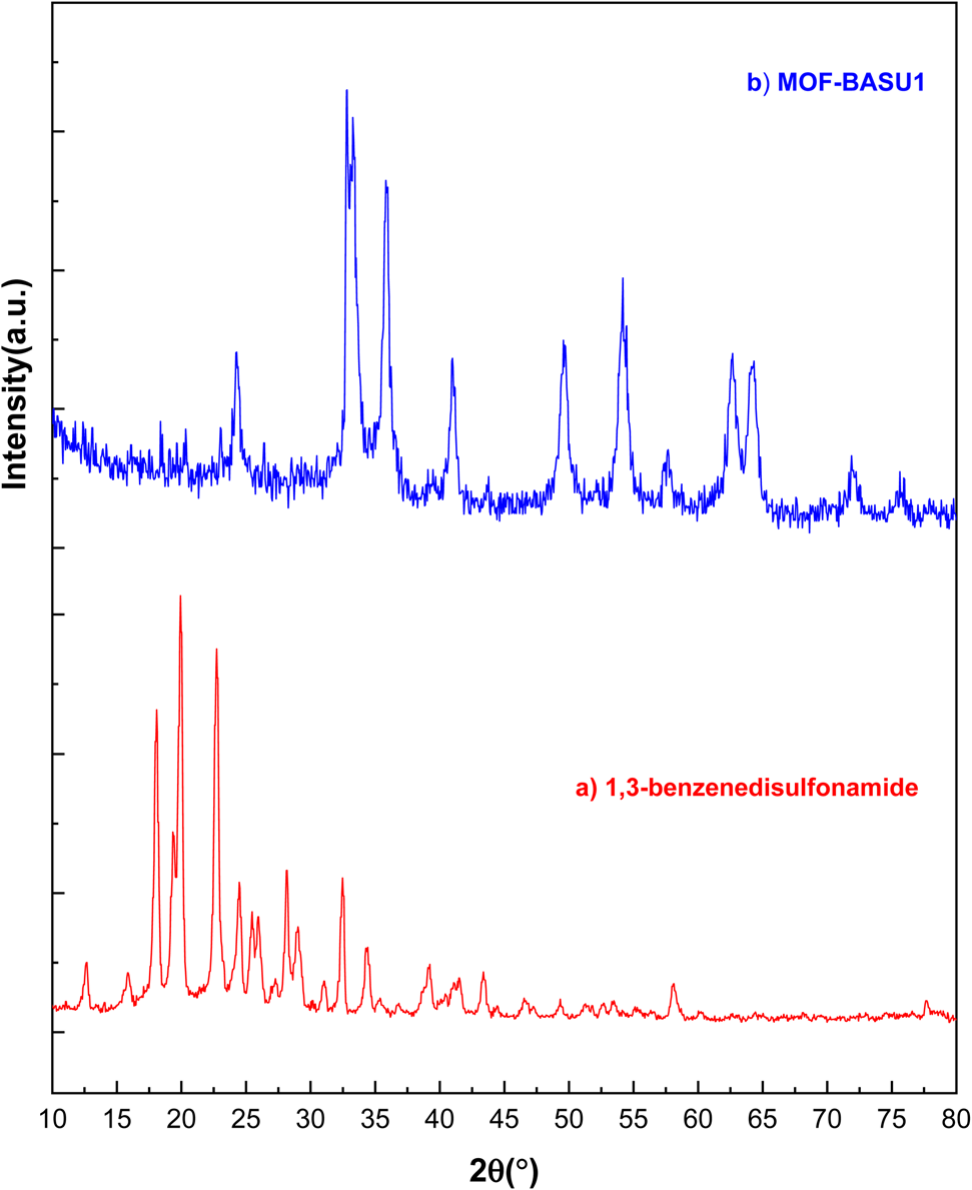
P-XRD image of 1,3-benzenedisulfonamide (a), and MOF-BASU1 samples (b).

XRD analysis was used to check the crystal structures of 1,3-benzenedisulfonamide and MOF-BASU1 samples (Fig. 2). The XRD spectrum of 1,3-benzenedisulfonamide shows diffraction peaks at 2*θ* = 18°, 23°, 28°, 32°, and 40° (Fig. 2A). Also, the same peaks in the MOF-BASU1 sample were shifted compared to the pure 1,3-benzenedisulfonamide and appeared with less intensity (Fig. 2B). Moreover, the introduction of the iron into the MOF-BASU1 was well defined by the peaks at 52.46°, 58.40°, and 65.61° suggesting the clear growth in the crystal structure. The average distance and crystal size were then calculated by Scherrer and Bragg equations to determine 45 nm.

**Fig. 3:**
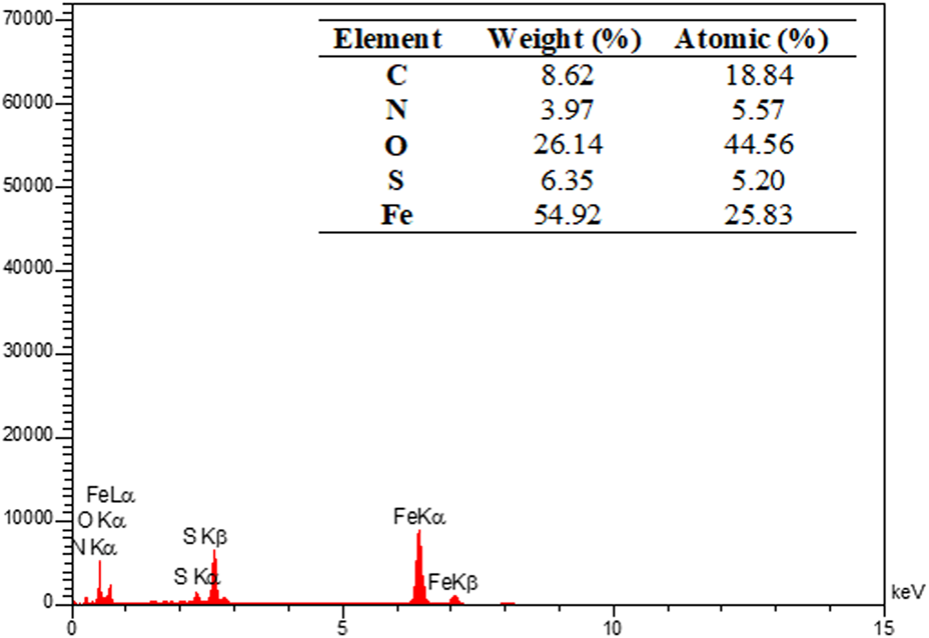
SEM-EDX micrograph of MOF-BASU1 catalyst.

**Fig. 4:**
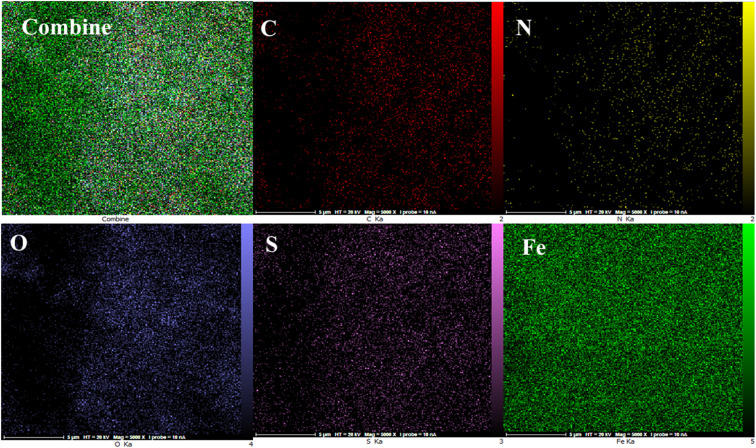
Elemental analysis of C, N, O, S, and Fe atoms achieved from SEM micrographs.

The energy-dispersive X-ray spectroscopy (EDX) elemental mapping of C, N, O, S, and Fe in MOF-BASU1 is depicted in Fig. 3. The analysis exhibited that all the required elements, including C (31.66%), O (22.10%), N (15.69%), S (8.55%), and Fe (3.46%) are present in the structure of MOF-BASU1. The elemental mapping studies of MOF-BASU1 show a uniform distribution of carbon, oxygen, iron, nitrogen, and sulfur components in the fabricated structure (Fig. 4).

**Fig. 5:**
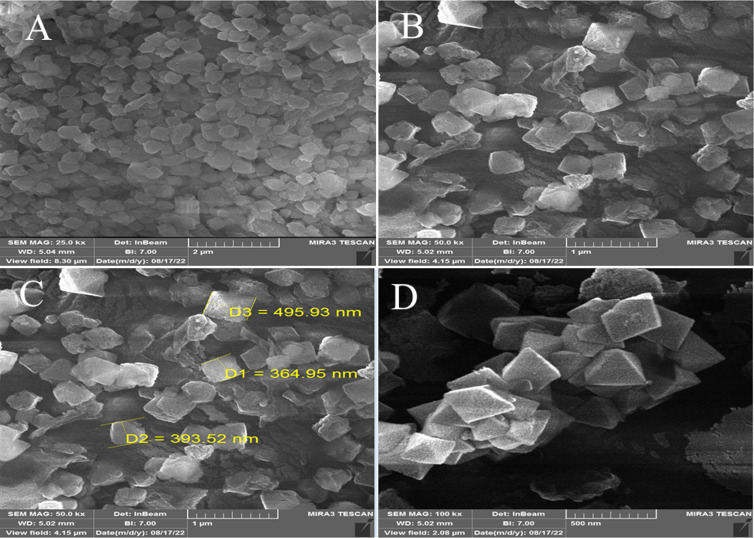
FE-SEM micrograph of MOF-BASU1 catalyst (A–D).

Fig. 5 shows the FE-SEM characterization of the size, shape, and morphology of the synthesized MOF-BASU1 nanocomposite. In the as-synthesized MOF-BASU1, we observed well distributed nearly-spherical uniform nanostructures (Fig. 5–D).

**Fig. 6:**
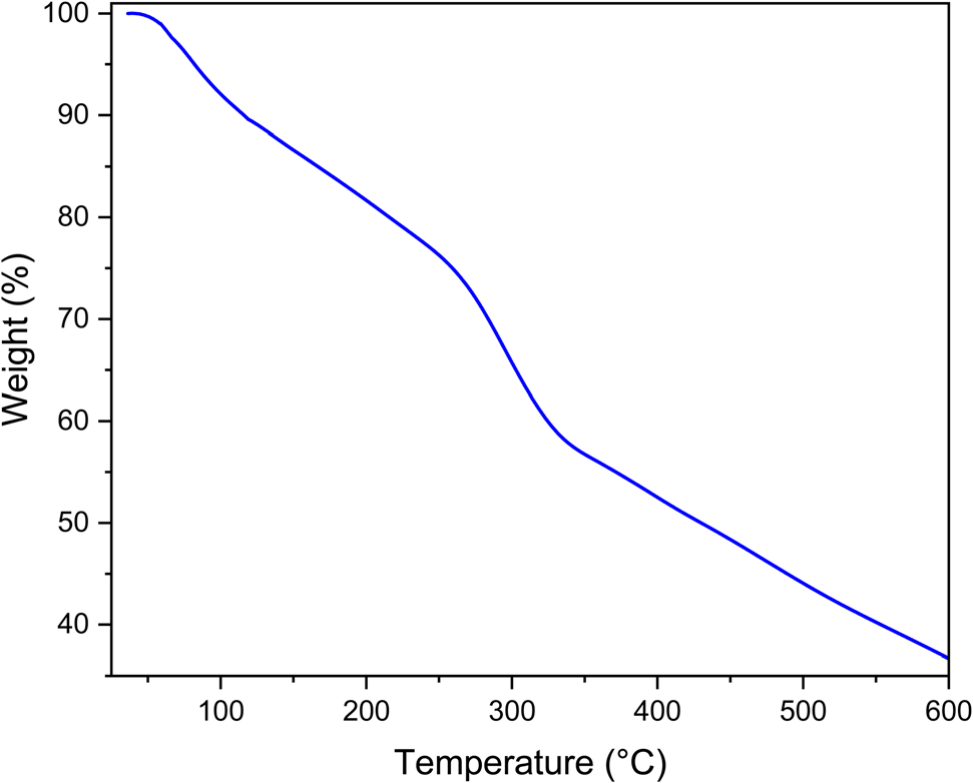
TGA spectrum of MOF-BASU1 catalyst.

Fig. 6 shows the TGA carried out to study the stability of the prepared catalyst. The first step initiates from 25 to 200 °C. This step is attributed to solvent removal. According to Fig. 6, MOF-BASU1 decomposes at 309.14 °C, and generally loses 37.59% of its weight.

**Fig. 7:**
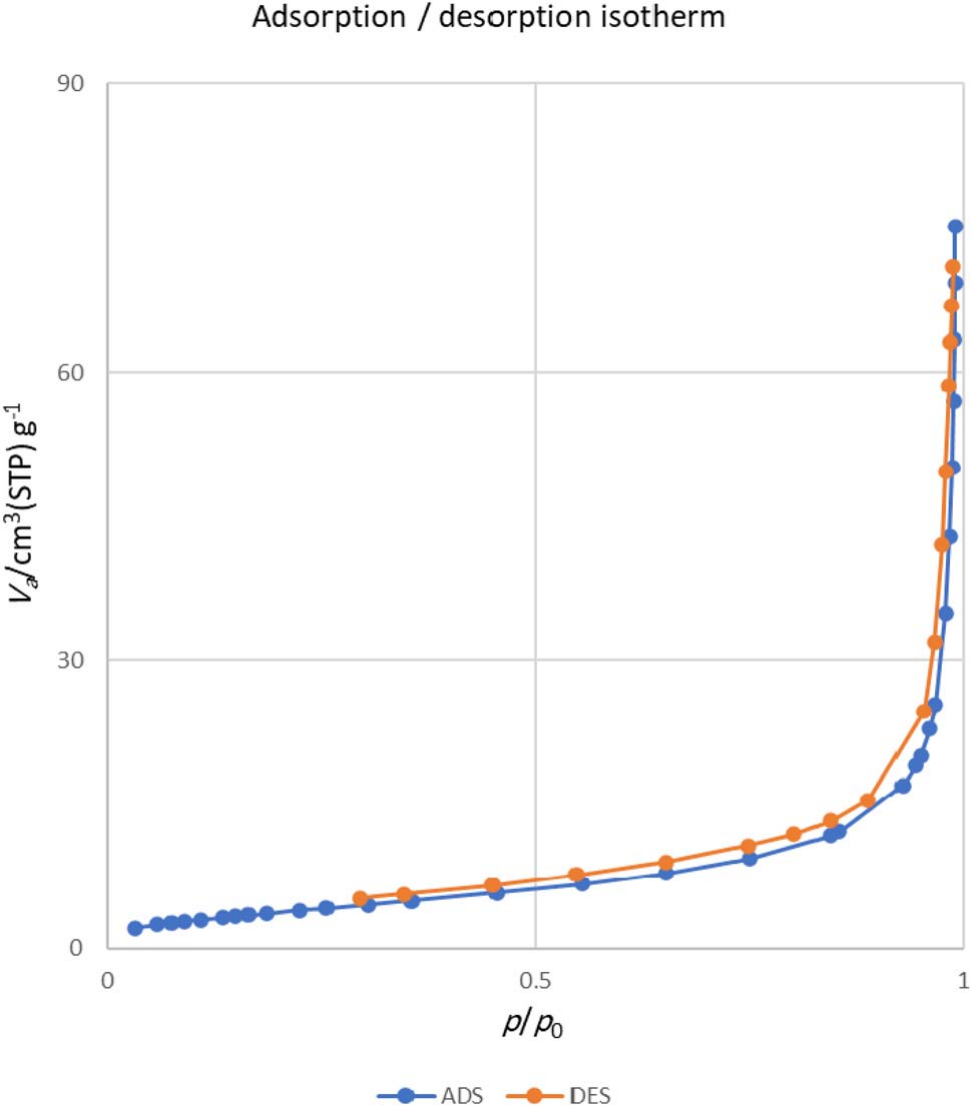
N_2_ adsorption and desorption isotherms of MOF-BASU1.

The important parameters associated with the porosity of MOF-BASU1 sample, including the surface area, average diameter of the pores, and total pore volume of the pores, are reported in Table 2. Fig. 7 indicates the N_2_ adsorption isotherms of the sample. Nitrogen adsorption and desorption curves of the sample show type I isotherm with H_2_ hysteresis curves, which confirms their microporous structure.

**Table 2:** Structural data of the MOF-BASU1

Sample	Surface area (m^2^ g^−1^)	Mean pore diameter (nm)	Total pore volume (cm^3^ g^−1^)
MOF-BASU1	14.56	30.09	0.11

### 3.2. Optimization conditions

To optimize the reaction parameters for the best catalytic property, the oxidation reaction of *p*-chlorobenzyl alcohol was used as a model reaction, and several reaction conditions, such as the presence and absence of different catalysts, solvents, and catalyst concentrations, were investigated (Table 3). First, the experimental reaction was carried out without a catalyst for 12 hours under an acetonitrile:water solvent system (1 : 1, volume), and no product was generated at room temperature (entry 1). Next, the temperature of the reaction is a further area for optimization, and we increased the temperature from room temperature to reflux, but no reaction progressed (entry 2). The same reaction was subsequently tested with TBHP oxidant, in an attempt to improve the catalyst activity. No reaction was progressed at reflux condition for a prolonged period of time (entry 3). This proved that the catalyst was necessary for carrying out these reactions. The same reaction was then conducted using a variety of MOFs, including IRMOF-3, basolite (Fe), MIL (101)Fe, and UiO-66-NH_2_ in TBHP. However, even after a 6 hours reaction, these catalysts only produced modest yields (entries 4–7). Encouraged by the above results, and in an endeavor to enhance the efficiency of the pilot reaction, the reaction was repeated using MOF-BASU1. Remarkably, the reaction took 1 hour to reach a high product yield of 97% (entry 8). Next, the solvent plays a vital influence in chemical conversions in terms of reaction time and product yield. The impact of several solvents and solvent-free conditions on the pilot reaction was investigated (entries 9–14). The oxidation reaction did not proceed in toluene because of the low dispersion of the synthesized catalyst in toluene (entry 9). While utilizing different solvents, such ethanol, acetonitrile, and DMF, under identical reaction conditions led to higher yields. Surprisingly, the same reaction, when conducted in a mixture of H_2_O : CH_3_CN (1 : 1, volume), produced a notable result due to the catalyst surface's enhanced activity. The typical reaction was explored for the different catalytic loadings of MOF-BASU1 under moderate reaction conditions in the presence of H_2_O : CH_3_CN solvent (1 : 1, volume) in order to improve reaction conditions for the influence of catalyst loading (entries 16–17). After investigating the reaction with 7 mg to 20 mg of catalyst, the product yields increased and reaction time decreased. Next, utilizing more than 15 mg of catalyst did not result in any appreciable improvements in yield or reaction time. Consequently, the ideal catalyst material, MOF-BASU1 (15 mg) (3 mg of iron from inductively coupled plasma (ICP) analysis. 0.075 mmol of Fe) was used to provide the desired result in 60 minutes reaction time. Finally, the same reaction was then conducted at 60 °C in H_2_O : CH_3_CN (1 : 1, volume). However, even after a 6 hours reaction time, the catalyst only produced modest yields (entry 18). It is important to note that superior results were achieved using pure oxygen (entry 19) (10 hours, 80%). It should also be noted that using less than 3 equivalents does not provide significant improvements in the efficiency or reaction time (entry 20).

**Table 3:** Optimization of the reaction conditions^a^

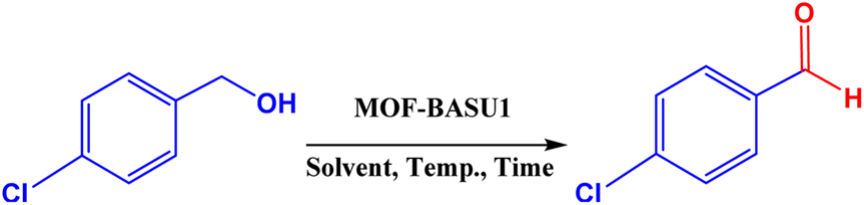						
Entry	Catalyst (mg)	Solvent	Temperature (°C)	Time (h)	TBHP (mmol)	Yield^b^ (%)
1	—	H_2_O : CH_3_CN (1 : 1)	r.t.	12	—	N.R.
2	—	H_2_O : CH_3_CN (1 : 1)	80	12	—	N.R.
3	—	H_2_O : CH_3_CN (1 : 1)	80	12	3	Trace
4	IRMOF-3 (15)	H_2_O : CH_3_CN (1 : 1)	80	6	3	30
5	UiO-66-NH_2_ (15)	H_2_O : CH_3_CN (1 : 1)	80	6	3	35
6	Basolite (Fe) (15)	H_2_O : CH_3_CN (1 : 1)	80	6	3	55
7	MIL (101)Fe (15)	H_2_O : CH_3_CN (1 : 1)	80	6	3	62
8	MOF-BASU1 (15)	H_2_O : CH_3_CN (1 : 1)	80	1	3	98
9	MOF-BASU1 (15)	Toluene	80	1	3	N.R.
10	MOF-BASU1 (15)	CH_3_CN	80	1	3	80
11	MOF-BASU1 (15)	H_2_O	80	1	3	40
12	MOF-BASU1 (15)	EtOH	80	1	3	65
13	MOF-BASU1 (15)	H_2_O : EtOH (1 : 1)	80	1	3	75
14	MOF-BASU1 (15)	Solvent-free	80	1	3	52
15	MOF-BASU1 (15)	DMF	80	1	3	39
16	MOF-BASU1 (7.5)	H_2_O : CH_3_CN (1 : 1)	80	1	3	82
17	MOF-BASU1 (20)	H_2_O : CH_3_CN (1 : 1)	80	1	3	97
18	MOF-BASU1 (15)	H_2_O : CH_3_CN (1 : 1)	60	1	3	79
19	MOF-BASU1 (15)^c^	H_2_O : CH_3_CN (1 : 1)	80	10	3	80
20	MOF-BASU1 (15)	H_2_O : CH_3_CN (1 : 1)	80	1	2	80

a Reaction condition: *p*-chlorobenzyl alcohol (1.0 mmol).

b Isolated yields.

c The reaction was investigated in the absence of O_2_.

**Scheme 3:**
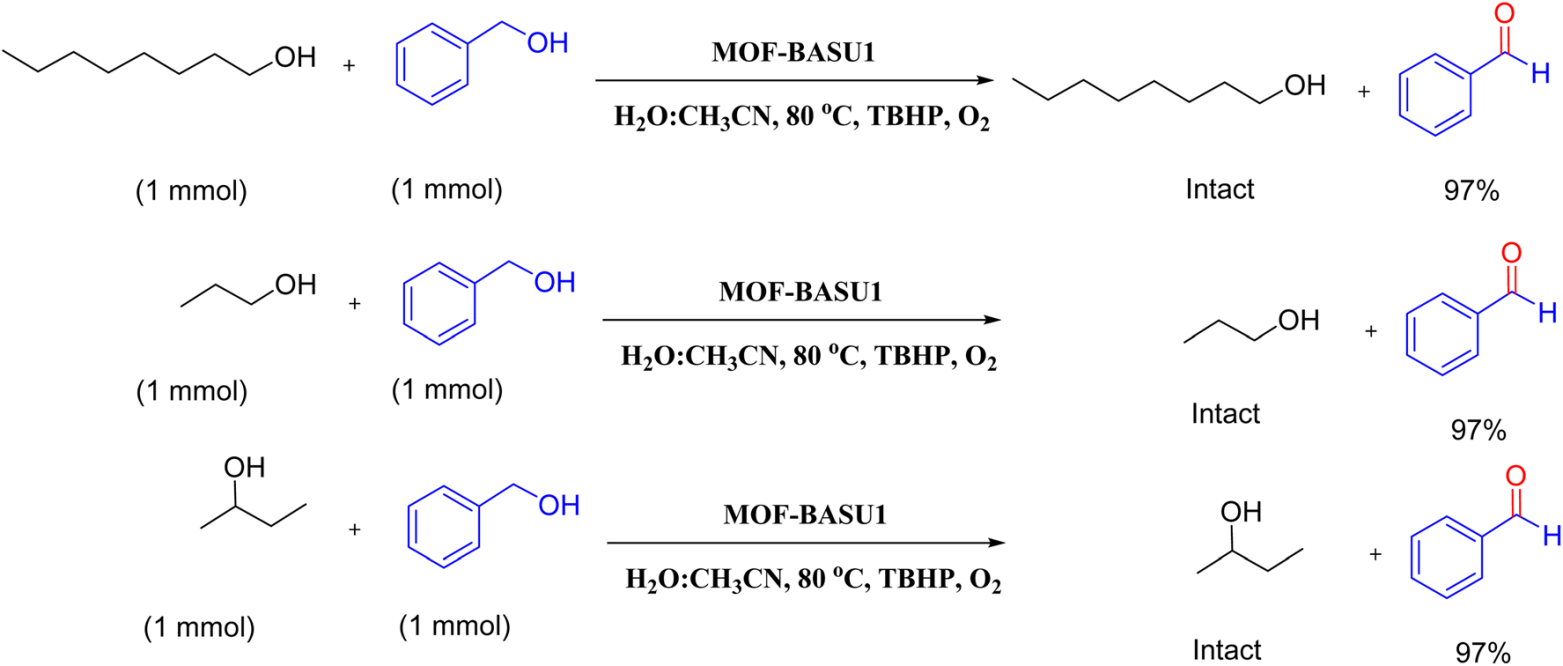
Intermolecular chemoselectivity tests.

Optimized conditions in hand, the generality and limitations of the catalytic activity of MOF-BASU1 were confirmed by developing the oxidation of benzyl alcohol derivatives. The nanocatalyst carried out the reactions chemoselectively with high yields. A range of functional groups on the phenyl ring of the benzyl alcohol, such as bromo, chloro, nitro, hydroxyl, methyl, and methoxy, were compatible under this procedure, and the products were isolated in good to high yields. Furthermore, when the reaction was performed with substrates bearing meta-substituted aryl rings, the reaction occurred with high selectivity (Table 4). The chemoselectivity of benzyl alcohol was investigated in the presence of aliphatic alcohols (Scheme 3). The results strongly confirmed the chemoselectivity of the present methodology for aldehydes.

**Table 4:** Oxidation of alcohols using MOF-BASU1^a^

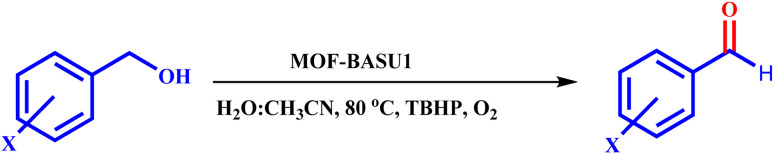							
Entry	Substrate	Product	Time (h)	Yield^b^ (%)	TOF^c^ (h^−1^)	TON^d^	Melting point–boiling point
1	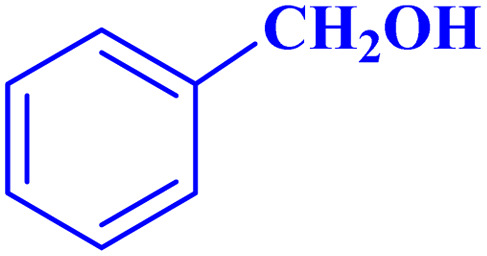	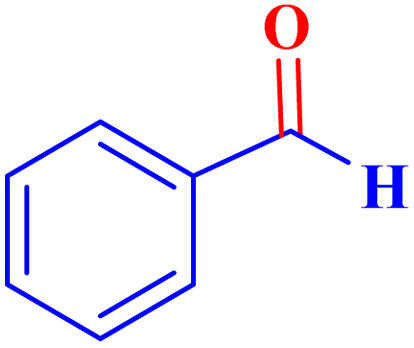	0.5	97	77.6	38.8	172
2	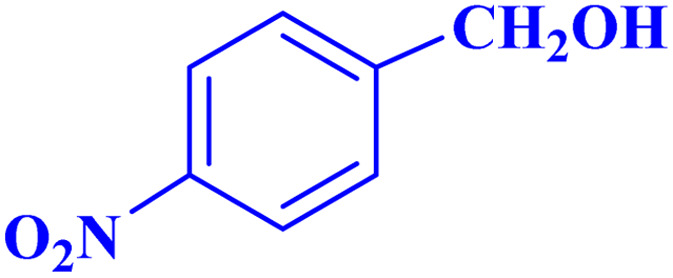	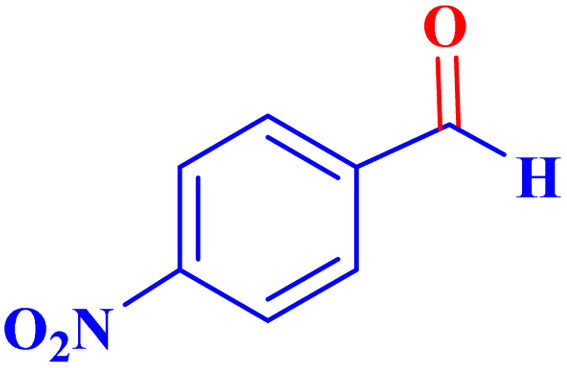	1.5	97	25.86	38.8	103–104
3	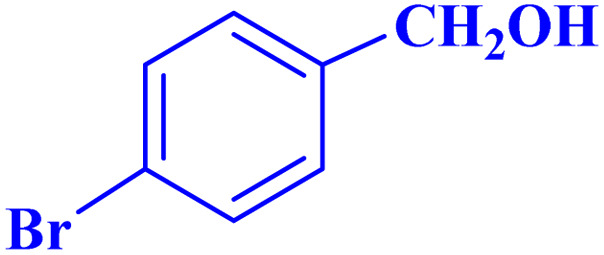	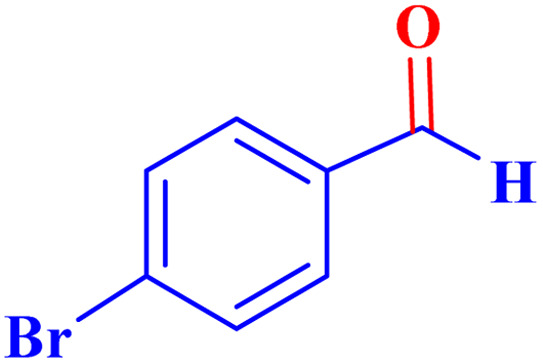	1.5	98	26.13	39.2	58–60
4	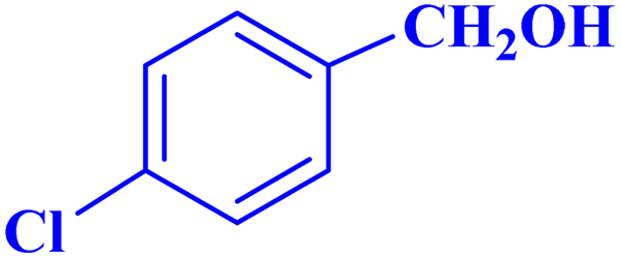	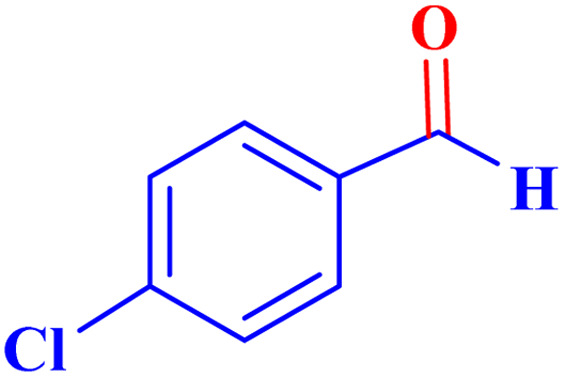	1	98	39.2	39.2	48–50
5	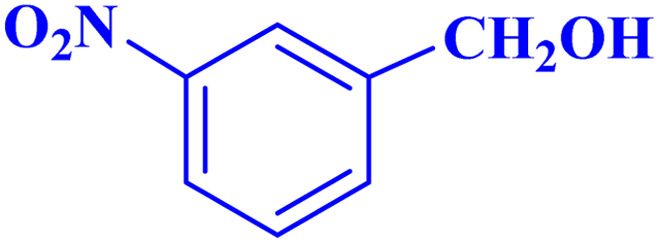	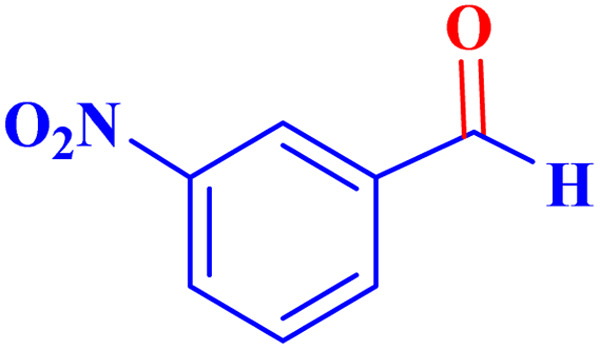	2	90	18	36	59
6	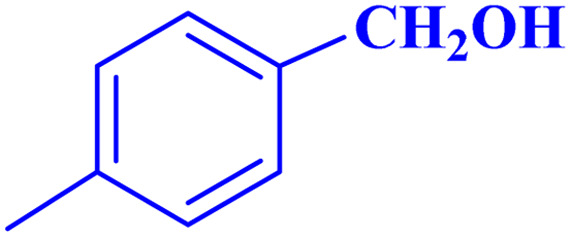	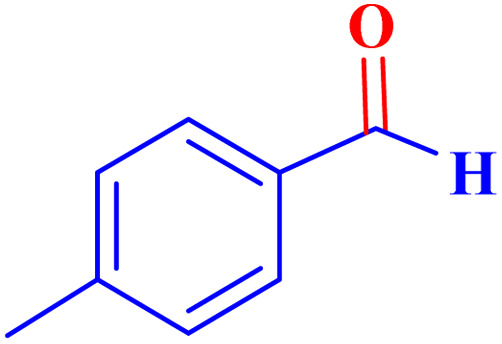	0.5	85	68	34	200
7	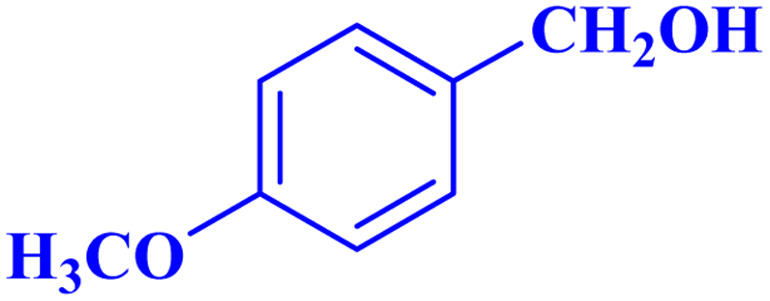	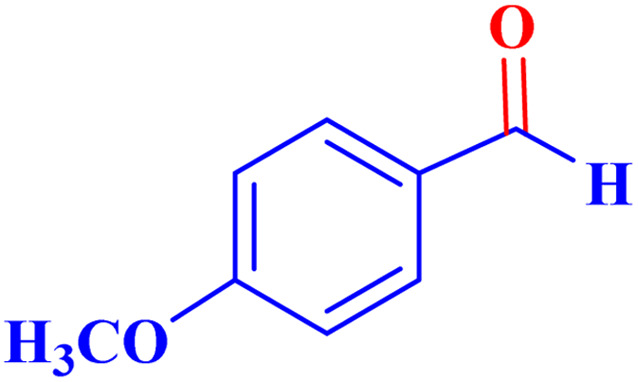	1	96	38.4	38.4	244–246
8	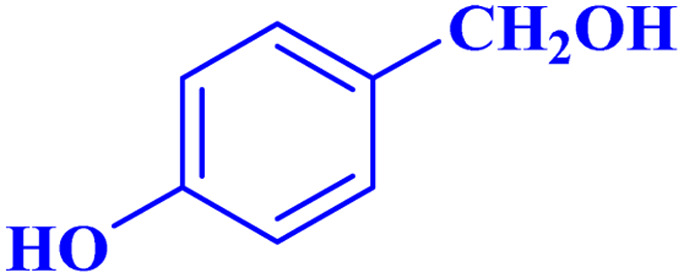	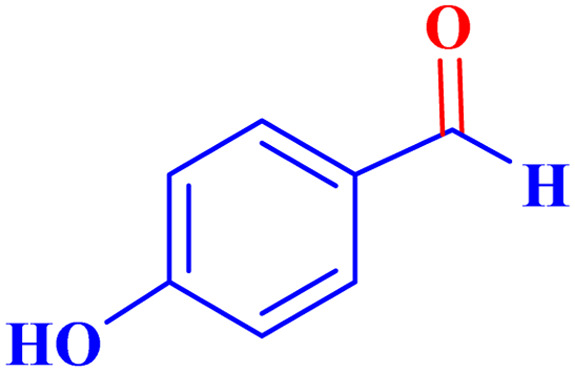	1.5	90	24	36	113–115
9	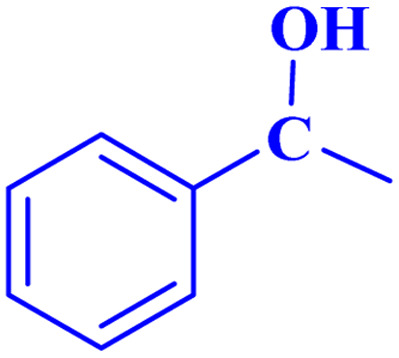	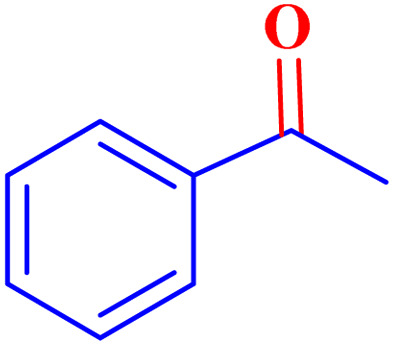	1	88	35.2	35.2	200
10	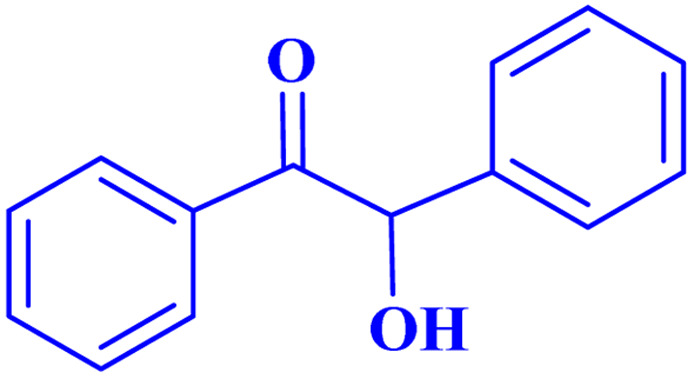	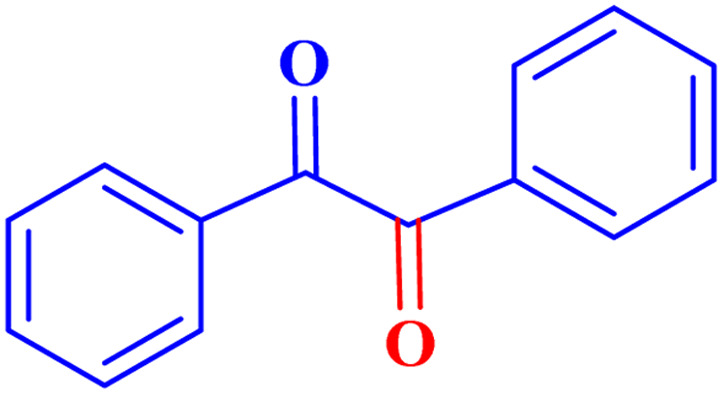	1	96	38.4	38.4	90–92

a Reaction condition: benzyl alcohol derivatives (1.0 mmol), TBHP (3 mmol).

b Isolated yields.

c TOF (turnover frequency) = TON per time (h).

d TON = yield (%)/catalyst (mol%).

In addition to catalyst activity, its reusability is a significant point in practical application. To investigate its recovery performance, MOF-BASU1 was examined by successive cycles of catalytic oxidation reactions. In a general experiment, the catalyst was isolated from an as-completed reaction solution and used directly for the next reaction cycle. Results of these studies exhibited that the catalyst could successfully be recycled and reused for 6 sequential runs without diminution in catalytic activity (98, 97, 96, 94, 92, and 90).

**Fig. 8:**
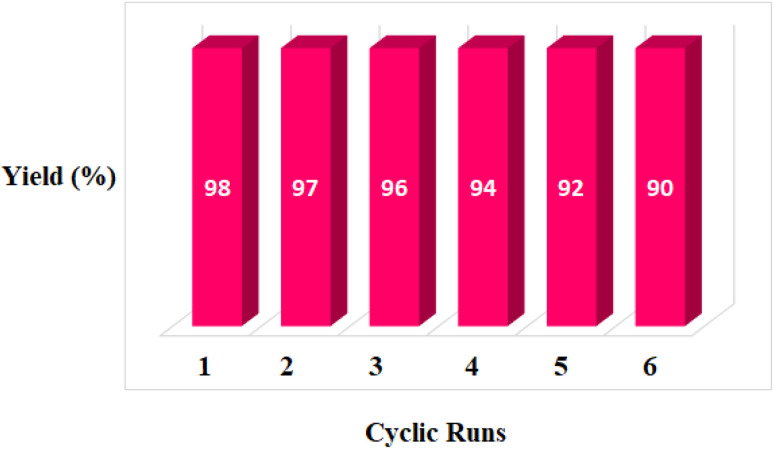
Recyclability of the MOF-BASU1 nanocatalyst for the oxidation of *p*-chlorobenzylalcohol.

According to a literature survey, the catalyst sites are the weak Brønsted acid sites, which may be attributed to uncoordinated sulfonamide groups, the redox-active,^31^ and Lewis acid iron sites.^32^ So, this catalyst can act usefully both as acid and redox active sites platform (Fig. 8).

**Scheme 4:**
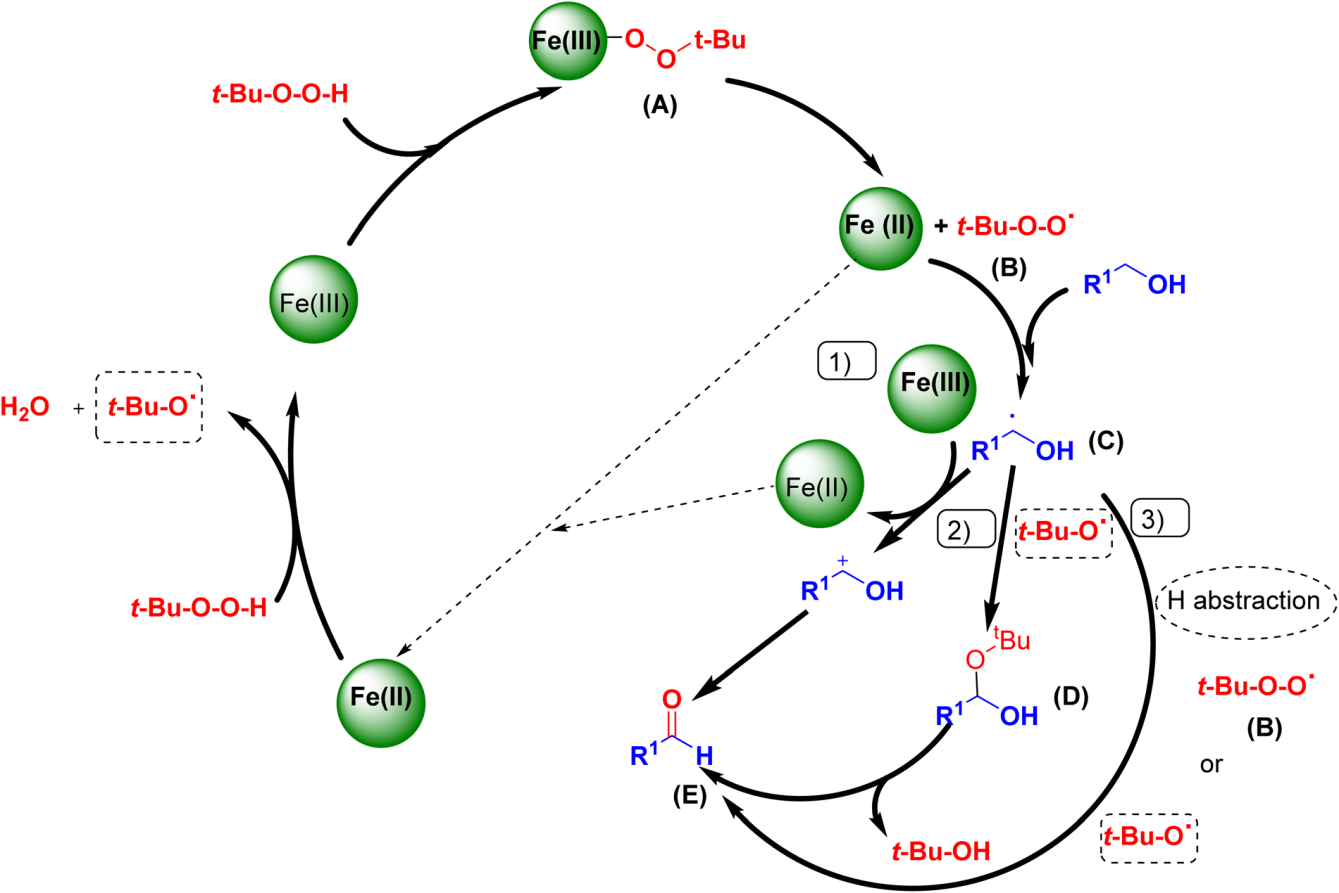
Mechanism of benzyl alcohol oxidation.

Based on the literature survey and the above argument, we introduce a mechanism for the oxidation process as shown in Scheme 4. At first, the coordination of *t*-BuOOH to the Fe(iii) sites affords *t*-Bu–OO–Fe(iii) species (A) which subsequently produces the active species (*t*-Bu–O–O˙(ii)). Then, the abstraction of a hydrogen-atom from the benzyl alcohol gives a radical intermediate (C). As the proposed mechanism shows, the three pathways: (1), (2), and (3) give the aldehyde product (E). The pathway (1) passes through the carbocation formation with the concomitant reduction of Fe(iii) to Fe(ii). The pathway (2) proceeds *via* a *gem*-diol-like structure formation and dehydration. The third pathway (3) involves a hydrogen-atom abstraction from (C) by the *t*-BuOO˙ and *t*-BuO˙ radicals.^33^ Moreover, H_2_ can be produced from methanol reforming in four different systems: MD, POM, SRM, and OSRM. Methoxy, formaldehyde, dioxymethylene, formate, and methyl formate are the commonly reported surface species detected on Cu-based catalysts.^34^

The advantages of the nanocomposites produced are summarized in Table 5 by comparing the results of the present study with the different catalytic systems used to produce aldehyde/ketone derivatives. According to these data, the catalytic system proposed herein, is more efficient in terms of reaction yield and time compared to other systems for this reaction. High yields and easy work-up are some advantages of this procedure (entry 7).

**Table 5:** Comparison of the catalytic performance of the MOF-BASU1 with other catalysts

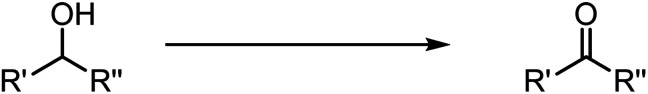					
Entry	Reaction condition	*T* (°C)	Time (h)	Yield (%)	Ref.
1	CuPc nanoparticle 5 mol%, *n*-Bu_4_NHSO_5_ (0.7 g), H_2_O	85	168	79	35
2	Fe(ii) phthalocyanines/TBHP (500/1)	70	3	94	36
3	Al-MCM-41(10)-CuPc (0.055 g)	100	4	47.5	37
4	CoPc@cell (0.05 g), KOH, *o*-xylene, O_2_	r.t.	8.5	83	38
5	MIL-101-NH_2_/CoTSPc (0.42 mol%), *p*-xylene, KOH, air (300 mL min^−1^)	100	8	95	39
6	TACoPc/Si–Cl (20 mg), H_2_O_2_, CH_3_CN, visible light	r.t.	6	60	40
7	MOF-BASU1 (20 mg), TBHP (3 mmol), CH_3_CN : H_2_O	80	1	98	This work

## 4. Conclusion

In this study, a novel iron-based MOF containing a sulfonamide (MOF-BASU1) was developed and synthesized for the oxidation of benzyl alcohol derivatives. Furthermore, the MOF-BASU1 nanocomposite was characterized as a multifunctional MOF with robustness and stability under reaction conditions. Hence, it can be reused several times without losing its catalytic activity and structural integrity. Other advantages of the proposed mesoporous catalyst include high efficiency, easy separation of the catalyst from the mixture, and clean reaction profile. This research depicts a bright future in the use of porous MOFs and their functionalized analogs as multifunctional catalysts.

## Author contributions

Mahtab Yaghubzadeh: doing laboratory work and preparing data. Sedigheh Alavinia: involved in conceptualization, methodology, resources, writing—original draft, reviewing and editing, formal analysis. Ramin Ghorbani-Vaghei: took part in conceptualization, investigation, supervision, writing—review and editing.

## Conflicts of interest

There are no conflicts to declare.

## Supplementary Material

RA-013-D3RA03058J-s001
