# Correction: A sustainable protocol for selective alcohols oxidation using a novel iron-based metal organic framework (MOF-BASU1)

**DOI:** 10.1039/d4ra90122c

**Published:** 2024-10-10

**Authors:** Mahtab Yaghubzadeh, Sedigheh Alavinia, Ramin Ghorbani-Vaghei

**Affiliations:** a Department of Organic Chemistry, Faculty of Chemistry, Bu-Ali Sina University Hamedan Iran rgvaghei@yahoo.com ghorbani@basu.ac.ir +98-8138380647

## Abstract

Correction for ‘A sustainable protocol for selective alcohols oxidation using a novel iron-based metal organic framework (MOF-BASU1)’ by Mahtab Yaghubzadeh *et al.*, *RSC Adv.*, 2023, **13**, 24639–24648, https://doi.org/10.1039/D3RA03058J.

The authors regret that Scheme 1 was not the best version and did not show the coordination of iron in the MOF clearly. An improved version of Scheme 1 is presented below:

**Scheme 1:**
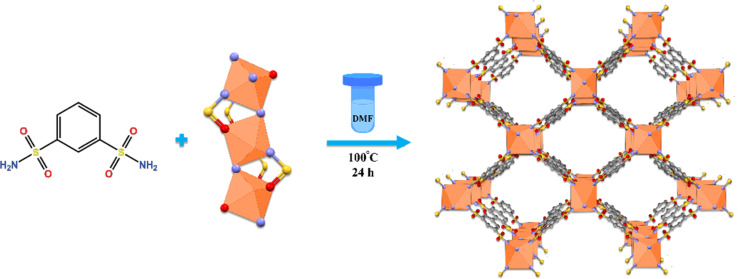
Sequential synthesis of MOF-BASU1 catalyst.

The authors regret an incorrect version of Fig. 1 was included in the original article. The correct version of Fig. 1 is presented below:

**Fig. 1:**
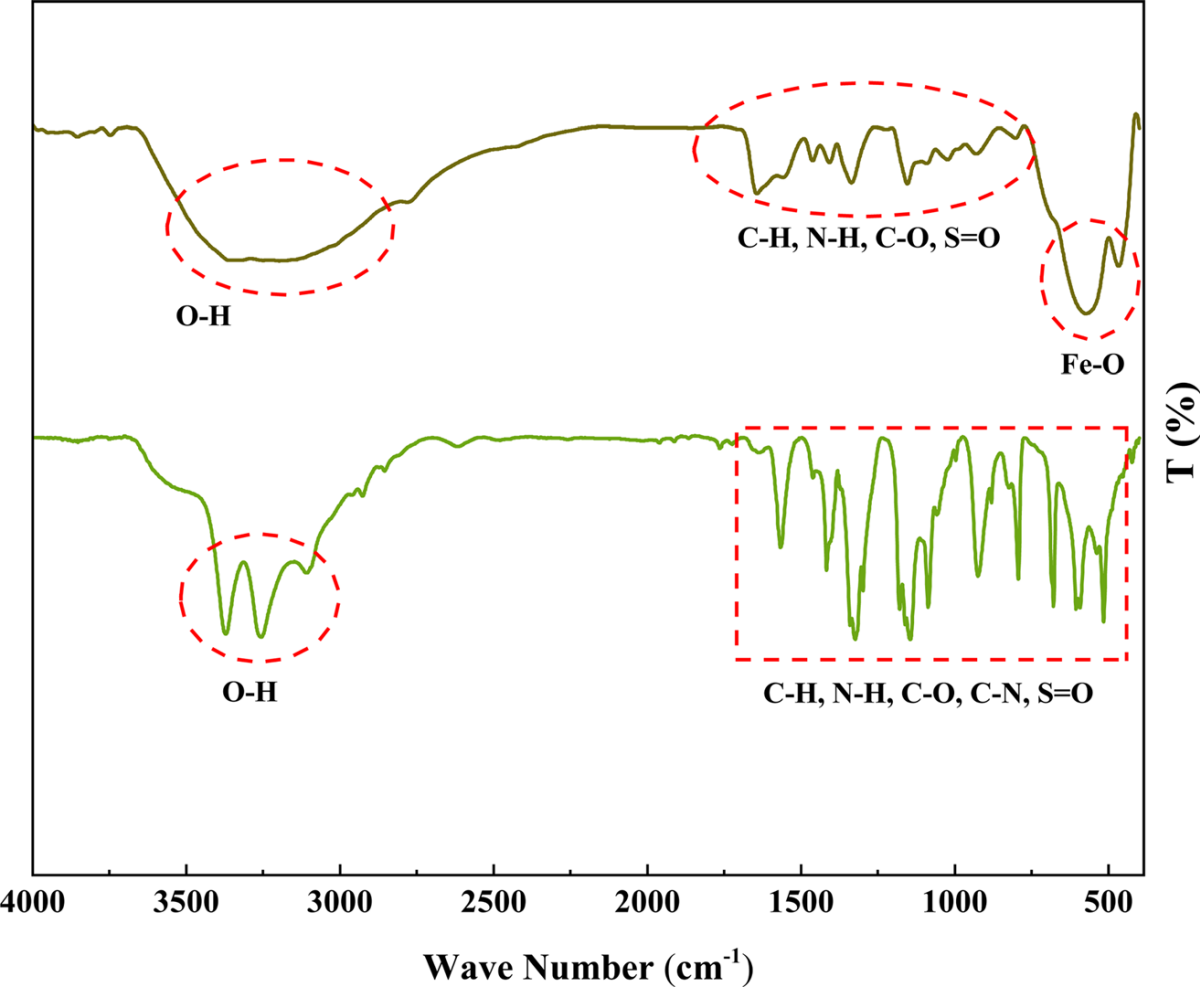
FT-IR spectra of 1,3-benzenedisulfonamide (A) and MOF-BASU1 samples (B).

In addition, the text accompanying Fig. 3 in the section “3.1. Catalyst characterization data analysis” should have read:

The analysis exhibited that all the required elements, including C (8.62%), O (26.14%), N (3.97%), S (6.35%), and Fe (54.92%) are present in the structure of MOF-BASU1. The elemental mapping studies of MOF-BASU1 show a uniform distribution of carbon, oxygen, iron, nitrogen, and sulfur components in the fabricated structure (Fig. 4).

The authors regret that in the version of Fig. 5 in the original article the same image was repeated in Fig. 5B and 5C, with Fig. 5C showing the particle sizes. To avoid confusion, a new version of Fig. 5 without the duplication is shown below:

**Fig. 5:**
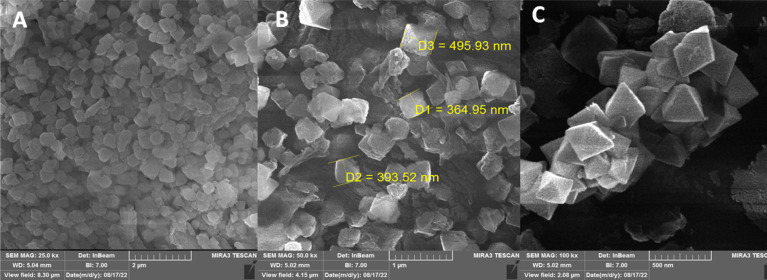
FE-SEM micrograph of MOF-BASU1 catalyst (A–C).

The Royal Society of Chemistry apologises for these errors and any consequent inconvenience to authors and readers.

