# Supplementary material for: A sustainable protocol for selective alcohols oxidation using a novel iron-based metal organic framework (MOF-BASU1)
Source: RSC Adv. 2023 Aug 17;13(35):24639–48. doi: 10.1039/d3ra03058j (PMC10433720; doi:10.1039/d3ra03058j)
Supplement: RA-013-D3RA03058J-s001 [file RA-013-D3RA03058J-s001.pdf]

Electronic Supplementary Material (ESI) for RSC Advances.
This journal is © The Royal Society of Chemistry 2023

A sustainable protocol for selective alcohols oxidation using a novel iron-based metal organic framework (MOF-BASU1)

Mahtab Yaghubzadeh, Sedigheh Alavinia, Ramin Ghorbani-Vaghei*
Department of Organic Chemistry, Faculty of Chemistry, Bu-Ali Sina University, Hamedan, 6517838683, Iran
*Corresponding author; E-mail: rgvaghei@yahoo.com&ghorbani@basu.ac.ir
Tel: +98(81)38380647

Spectral data for products.

^{1}H NMR (250 MHz, DMSO-d_6) δ 9.99 (s, 1H), 7.90 (dd, *J* = 6.3, 5.2 Hz, 2H), 7.54 (d, *J* = 7.6 Hz, 2H), ^{13}C NMR (63 MHz, DMSO-d_6) δ 192.81, 166.89, 138.18, 131.57, 129.16.

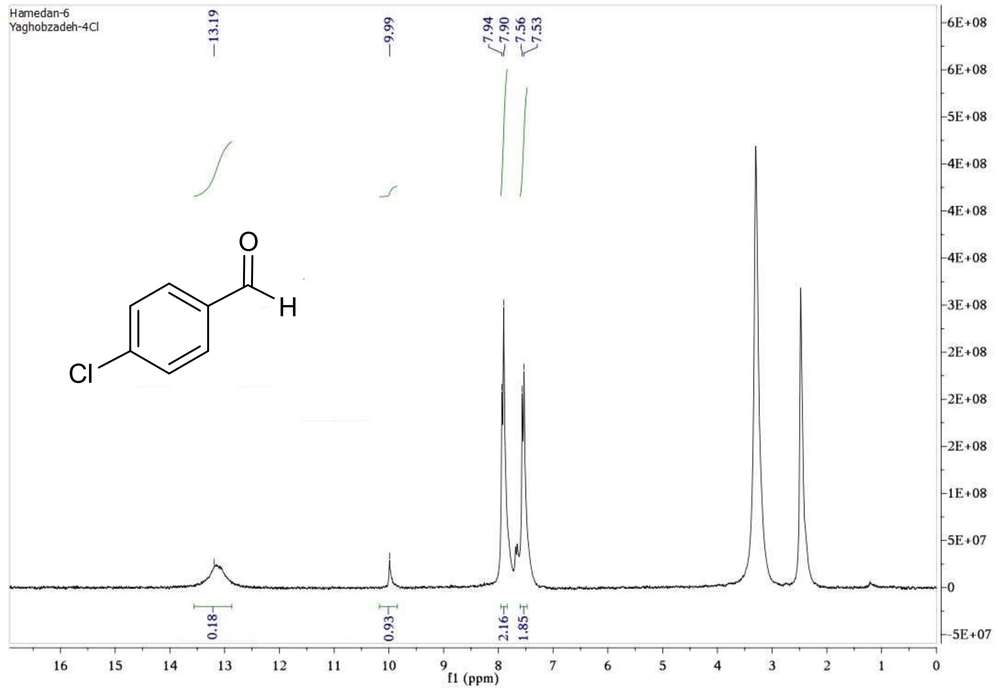

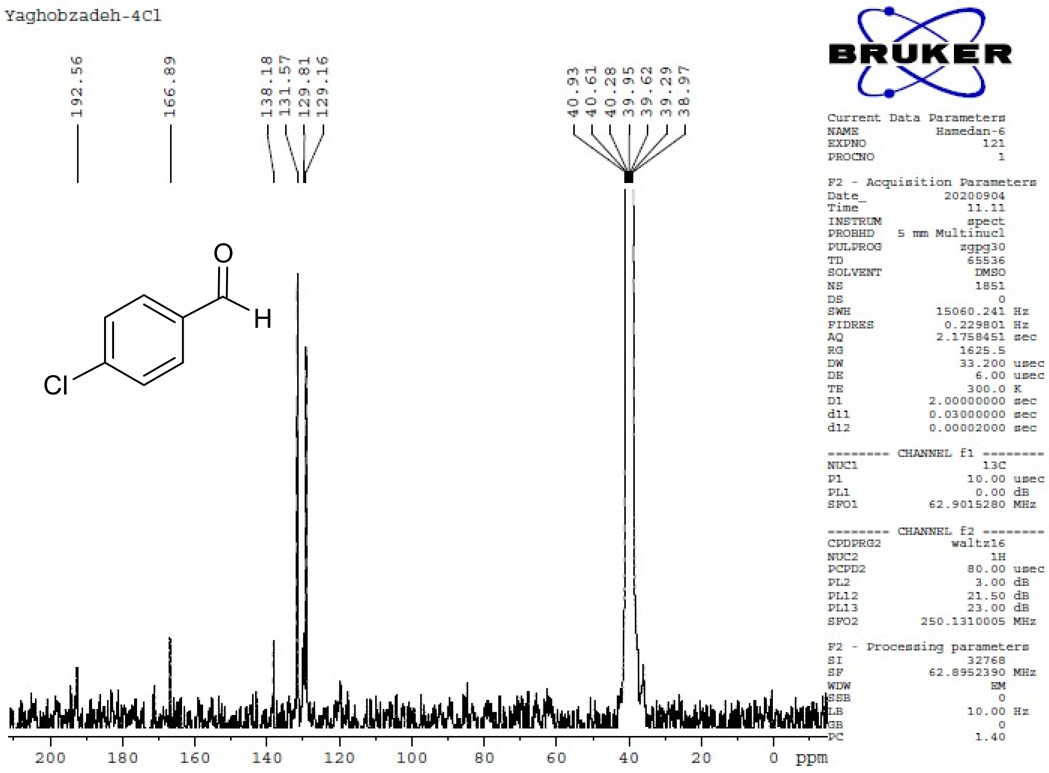

^{1}H NMR (250 MHz, DMSO-d_6) δ 10.11 (s, 1H), 8.65 (s, 1H), 8.49 (d, J = 6.2 Hz, 1H), 8.30 (d, J = 6.3 Hz, 1H), 7.97 – 7.78 (m, 1H), ^{13}C NMR (63 MHz, DMSO-d_6) δ 192.24, 148.69, 137.60, 135.32, 131.37, 128.95, 124.46.

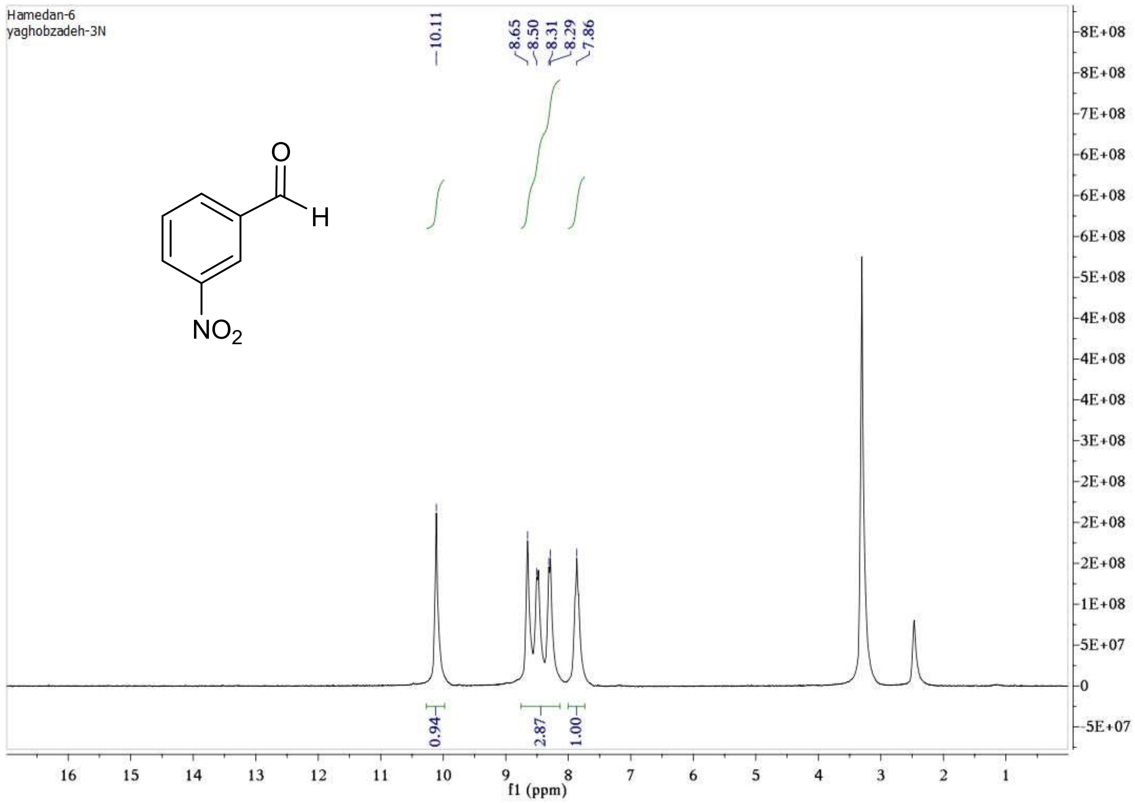

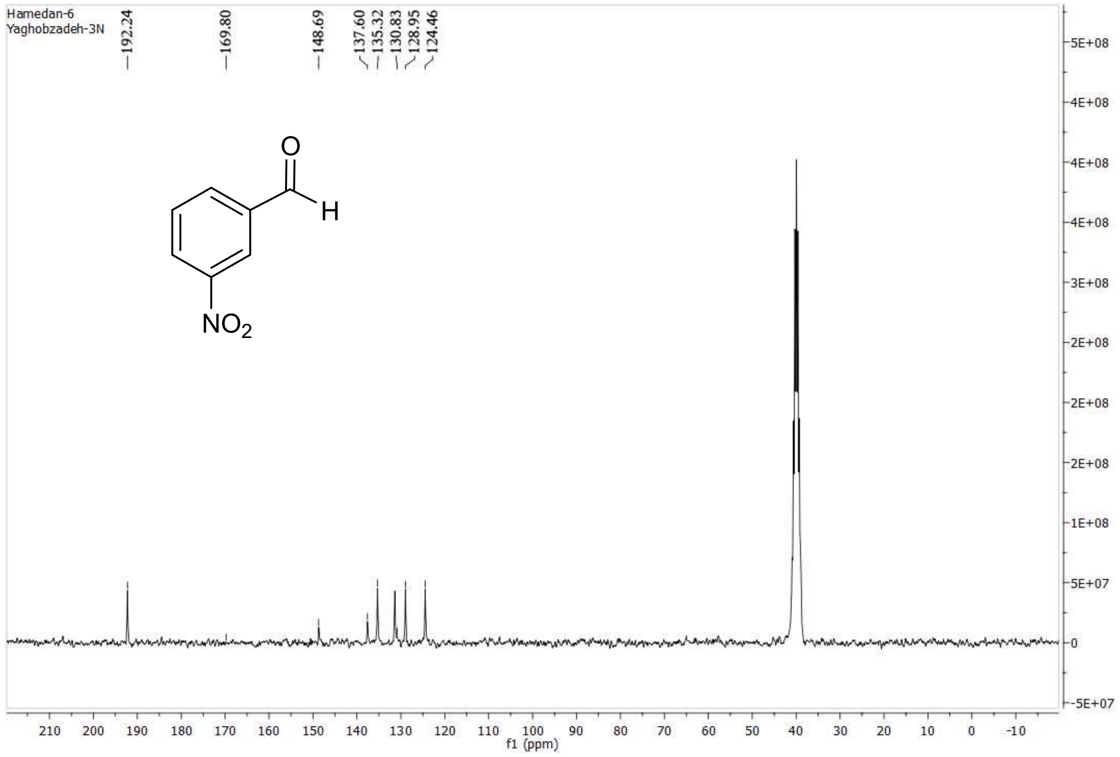

^{1}H NMR (250 MHz, DMSO-d$_6$) δ 10.55 (s, 1H), 9.76 (s, 1H), 7.73 (d, *J* = 7.2 Hz, 2H), 6.90 (d, *J* = 7.2 Hz, 2H), ^{13}C NMR (63 MHz, DMSO-d$_6$) δ 191.36, 163.73, 132.50, 128.83, 116.25.

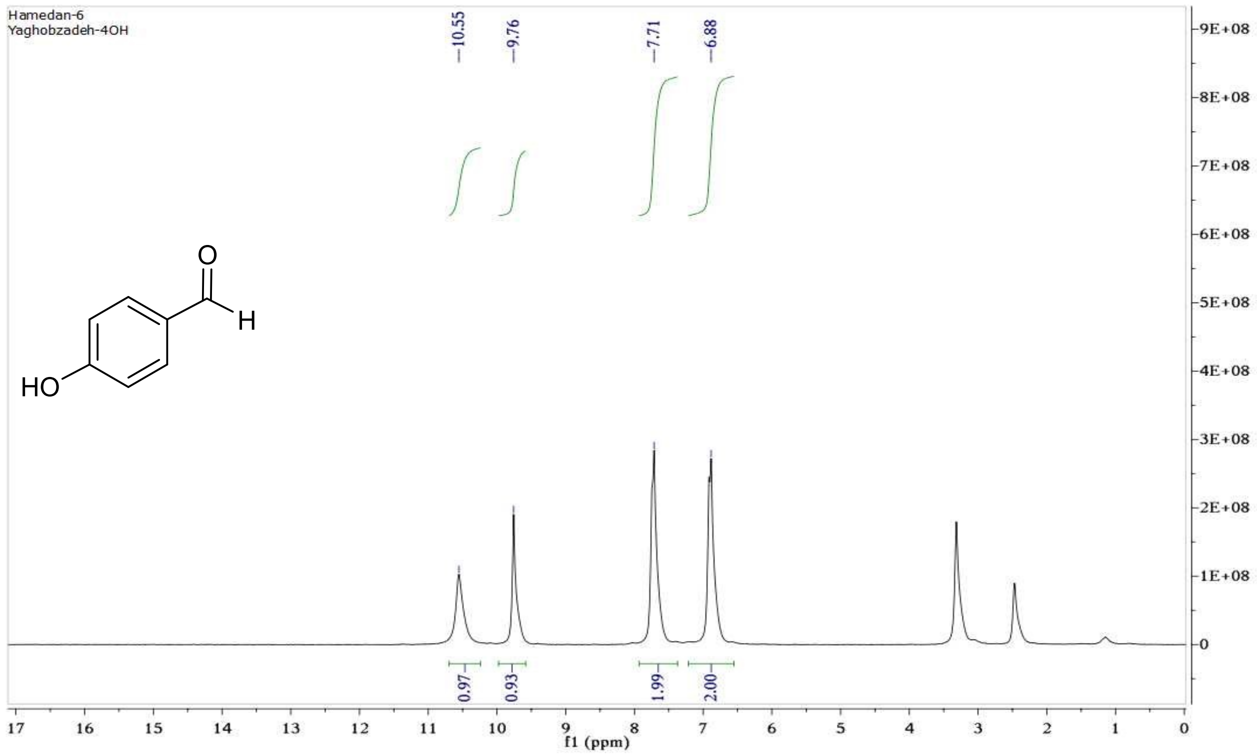

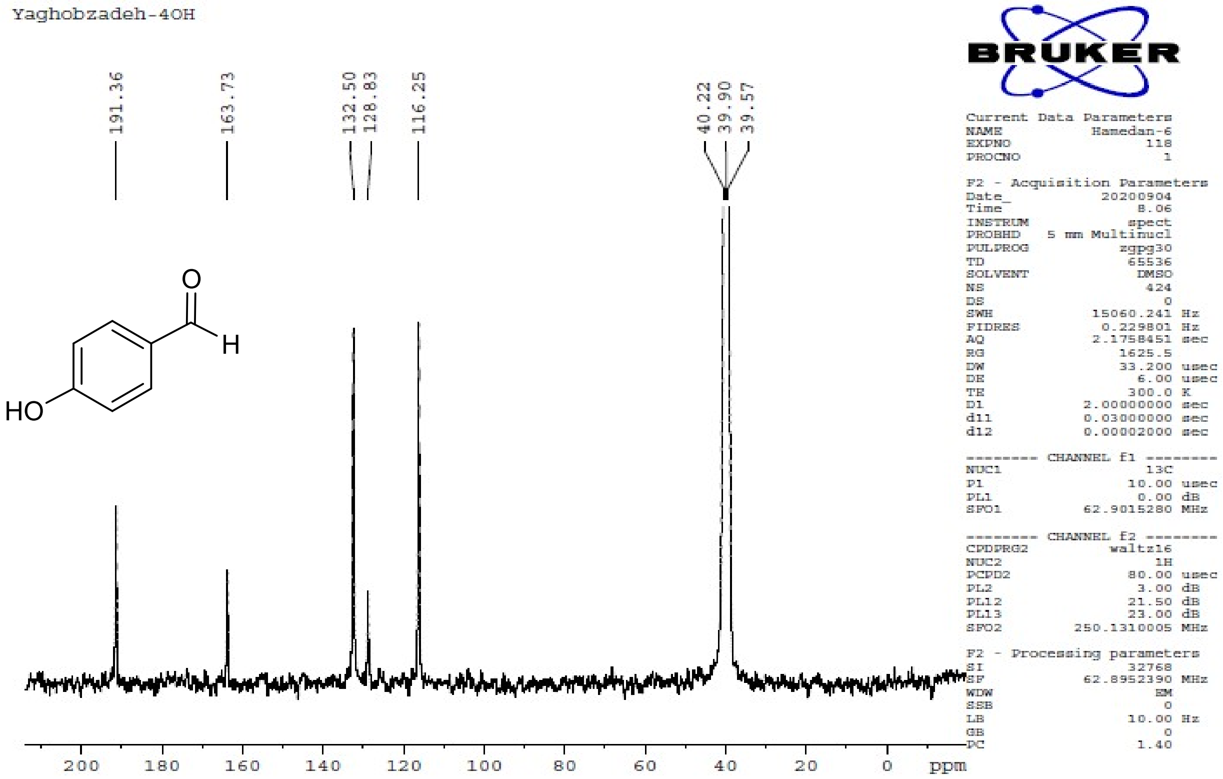

^{1}H NMR (250 MHz, DMSO-d_6) δ 10.13 (s, 1H), 8.37 (d, J = 6.8 Hz, 2H), 8.12 (d, J = 6.4 Hz, 2H), ^{13}C NMR (63 MHz, DMSO-d_6) δ 192.69, 140.25, 131.03, 124.66, 123.66.

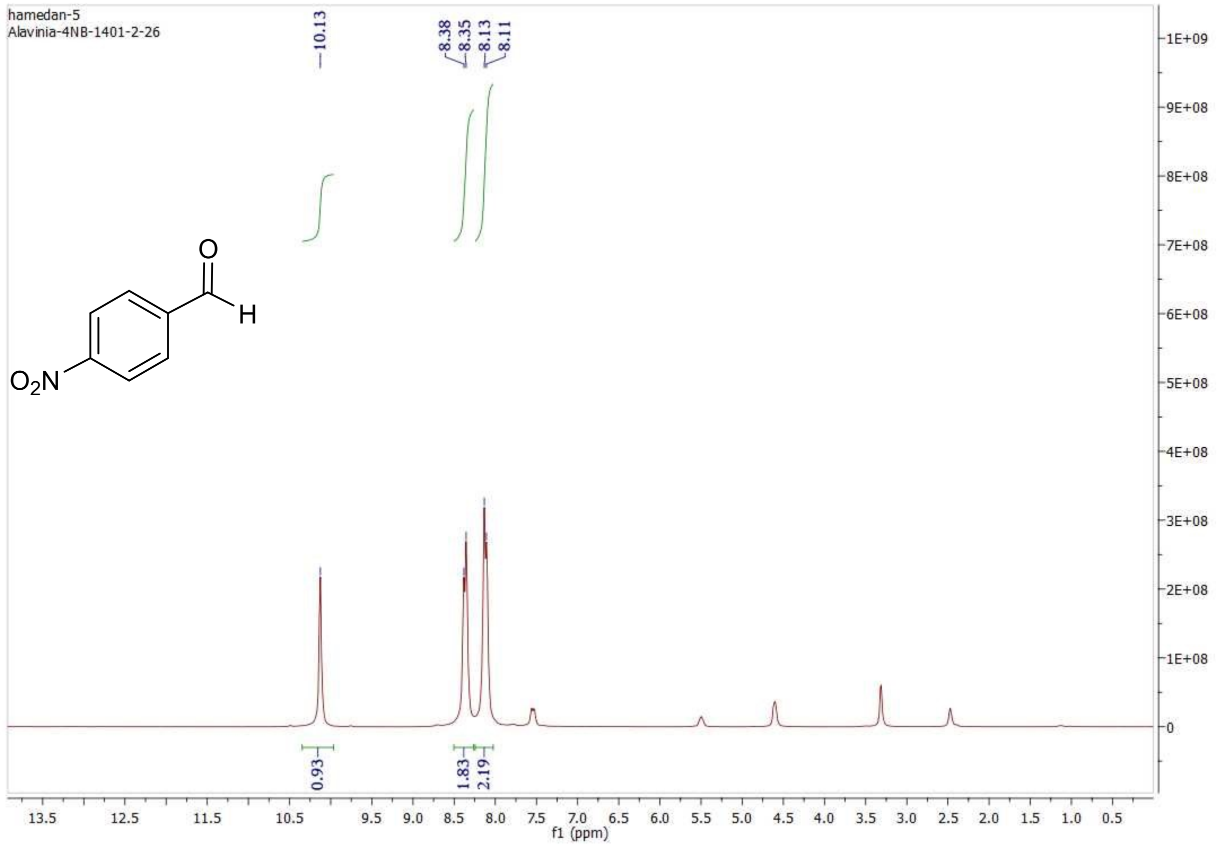
hamedan-5
Alavinia-4NB-1401-2-26
10.13
8.38
8.35
8.13
8.11
0.93
1.83
2.19
O
H
O_2N
f1 (ppm)

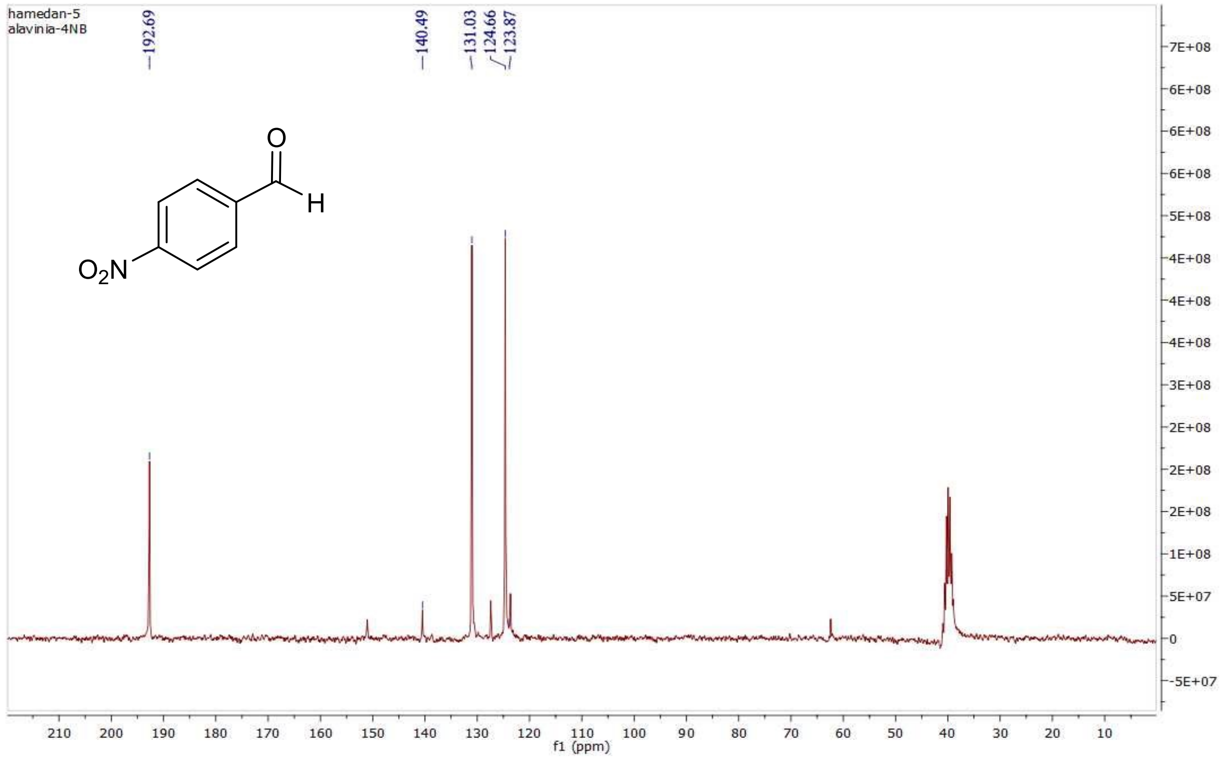
hamedan-5
alavinia-4NB
192.69
140.49
131.03
124.66
123.87
O
H
O_2N
f1 (ppm)
